# Modular Approach to Select Bacteriophages Targeting *Pseudomonas aeruginosa* for Their Application to Children Suffering With Cystic Fibrosis

**DOI:** 10.3389/fmicb.2016.01631

**Published:** 2016-10-13

**Authors:** Victor Krylov, Olga Shaburova, Elena Pleteneva, Maria Bourkaltseva, Sergey Krylov, Alla Kaplan, Elena Chesnokova, Leonid Kulakov, Damian Magill, Olga Polygach

**Affiliations:** ^1^Laboratory for Genetics of Bacteriophages, Department of Microbiology, I.I. Mechnikov Research Institute for Vaccines and SeraMoscow, Russia; ^2^Medical Biology Centre, School of Biological Sciences, Queen’s University BelfastBelfast, UK

**Keywords:** choice of phage, Phage therapy organization, cystic fibrosis, phage–host interactions, *Pseudomonas aeruginosa*, modular phage compositions, personalized phage therapy

## Abstract

This review discusses the potential application of bacterial viruses (phage therapy) toward the eradication of antibiotic resistant *Pseudomonas aeruginosa* in children with cystic fibrosis (CF). In this regard, several potential relationships between bacteria and their bacteriophages are considered. The most important aspect that must be addressed with respect to phage therapy of bacterial infections in the lungs of CF patients is in ensuring the continuity of treatment in light of the continual occurrence of resistant bacteria. This depends on the ability to rapidly select phages exhibiting an enhanced spectrum of lytic activity among several well-studied phage groups of proven safety. We propose a modular based approach, utilizing both mono-species and hetero-species phage mixtures. With an approach involving the visual recognition of characteristics exhibited by phages of well-studied phage groups on lawns of the standard *P. aeruginosa* PAO1 strain, the simple and rapid enhancement of the lytic spectrum of cocktails is permitted, allowing the development of tailored preparations for patients capable of circumventing problems associated with phage resistant bacterial mutants.

## Introduction

The use of antibiotics in the treatment of bacterial infections increasingly encounters difficulties caused by the emergence and rapid spread of pathogenic bacteria exhibiting multidrug resistance. The discovery and study of new antibiotics, is an extremely expensive and arduous process, associated with many risks (Fowler et al., 2014). As a result there is renewed interest in phage therapy – the use of bacterial viruses (phages), in their role as “natural enemies” of the bacteria. The main objective of phage therapy is the elimination of pathogenic bacteria within the foci of infection. But following the emergence and multiplication of phage-resistant bacteria, the only way forward will be through the use of new phages capable of overcoming this resistance.

Phage therapy was proposed and implemented for treating bacterial infections by Felix D’Herelle, one of the pioneers of bacterial viruses about 100 years ago. Affirmation as to the potential medical application of bacteriophages has been demonstrated by their prolonged use in the treatment of certain bacterial infections in Russia, Georgia, and Poland. Indeed, there is particular promise with respect to both the efficacy and safety of phage therapy toward superficial infections of skin and mucus membranes (Abedon et al., 2011; Krylov, 2014). In these countries therapeutic mixtures active against a variety of bacterial pathogens are produced on an industrial scale, so a significant level of expertise and a large collection of bacteriophages have been accumulated over the years. However, widespread acceptance of phage therapy is far from being achieved. The use of phage therapy in Western countries mostly ceased following the introduction of antibiotics into medical practice but now, due to the emergence of pathogenic bacteria resistant to all available antibiotics the need for a revival seems almost inevitable.

Scientific professionals are well aware of the epidemic potential of rapidly spreading multidrug resistance. This understanding has led to a detailed discussion regarding the need to implement the use of phage therapy in the medical practices of different countries and in providing solutions to the outstanding problems associated with such practices (Alavidze et al., 2016). Among them are problems of a legislative nature, the need to prove the long term safety of phage therapy, promoting the recognition of phage therapy beyond a handful of Eastern European countries to an international level, issues with the production of phage cocktails on an industrial scale, the financing associated with this, and problems with patenting.

Whilst the need to address these problems is recognized, one should bear in mind that the future will bring with it cases in which phages may be the only route to antibacterial therapy. An example of such a situation could involve the appearance of plasmids conferring bacterial resistance to colistin in specialized centers for the treatment of patients with cystic fibrosis (CF), resulting in a significantly worsened prognosis. Therefore, the problems associated with the implementation of phage therapy for the control of multidrug resistant *Pseudomonas aeruginosa* requires an immediate solution. In cases where repeated infections are inevitable (e.g., in CF patients), and no appropriate antibiotics are available, phage therapy may prove to be the only option to prolong the life expectancy of patients.

In parallel to this work, we believe in making the study of phage therapy in children suffering from CF a priority area. Success in such cases will secure a place for phages in our medical arsenal by establishing a positive public perception for the treatment along with the experience that will be accumulated in the course of the studies.

Here various aspects required for the implementation of phage therapy for *P. aeruginosa* pulmonary infection in children suffering from CF are discussed in detail. In the age of growing antibiotic resistance, phage therapy may prove to be the only feasible route to antibacterial therapy.

## Features of Cystic Fibrosis Due to Bacterial Infection

Cystic fibrosis is a frequent hereditary disease of Caucasians. Various estimates have placed on average one out of every 2500 – 7000 newborns as being homozygous for the mutant allele CFTRΔF508. The pathogenesis of the disease is associated with impaired secretory function of the pulmonary epithelium, leading to the disruption of ion exchange and accumulation of mucus and fluid in the lungs. This creates favorable conditions for the growth of bacteria of various species. At the early stages, staphylococcal infection is the major culprit, followed by *P. aeruginosa* domination (Kosorok et al., 2001). The production of alginate by these bacteria, an extremely viscous polysaccharide, by these bacteria promotes the proliferation and survival of other hazardous species, such as *Burkholderia sp.*, worsening the overall prognosis (Henry et al., 1992; Lynch and Dennis, 2012). Symptoms of this disease may differ significantly amongst individuals but the major ones tend to be obvious from a young age.

During the course of infection a gradual change in the properties of the primary infecting strains of *P. aeruginosa* takes place, manifested by a decrease in their pathogenicity and virulence, as well as increased sensitivity to the lytic effect of bacteriophages (Friman et al., 2013; Cullen et al., 2015). Moreover these strains, being adapted to the conditions of the lungs, are influencing the expression of the pathogenic properties of other species in the concomitant microflora (such as *Burkholderia multivorans, Burkholderia cenocepacia, Pandoraea pulmonicola* and *Pandoraea apista*) significantly lowering them.

It has been proposed that such domination is accomplished through gene products involved in quorum sensing and pyoverdine biosynthesis (Costello et al., 2014). The gross activity of the set of interacting bacteria leads to the destruction of the lungs, generally seen as the major reason for the reduction in life expectancy of CF patients. The use of new antibiotics as well as inhalation protocols for their introduction into lungs has significantly increased patient life expectancy. However, the use of antibiotics, even in high doses does not always lead to the eradication of *P. aeruginosa* due to the persistence of some bacterial cells through their transformation into dormant cyst-like and non-culturable cells (Mulyukin et al., 2015).

An important factor in the evolution of *P. aeruginosa* in specialized CF treatment centers is a constant inclusion of new bacterial strains carrying different prophages in their genomes, including transposable phages through cross-infection of patients. It is believed that the activity of the transposable phages in pathogenic islands has led to the emergence of strains exhibiting enhanced virulence, pathogenicity and resistance to environmental factors and, as a consequence, the capability for epidemic spread (Winstanley et al., 2009). Such epidemic spread is also a risk factor outside the CF centers (Mohan et al., 2008).

The different approaches were proposed to extend the life of patients and to improve its quality. Among them is the surgical replacement of patient’s lungs (Morrell and Pilewski, 2016). There was also hope to develop a drug for selective inhibition of alginate synthesis in bacterial cells (Hershberger et al., 1995). Another proposition was to substitute the function of the mutant gene, by creating a viral integratable vector carrying the wild-type allele (Yan et al., 2015). There is also a suggestion that modification of the activity of toll-like receptors and other coreceptors with genetic engineering may lead to changes in critical components of CF immunobiology. It is not yet possible, but is expected that with improvements in bioengeneering, that the development of novel vectors and methods of delivery, biocompatibility, and safety, then therapeutic effectiveness will be successfully achieved. This however, could take many years (Atkinson, 2008). Thus, one must assume that at the present time, and in the immediate future, antibacterial therapy will be the major approach to treat CF patients. This currently takes the form of “aggressive antibiotic therapy,” usually by alternating two antibiotics in order to reduce the probability of the occurrence and accumulation of multidrug-resistant mutants. In cases involving resistant strains arising, there is the option to utilize the inhalation of colistin as a last resort, a very toxic substance exhibiting a strong surface activity. Resistance to colistin due to mutations in the bacterial chromosome is a rare event because it requires mutations in two genes controlling the structure of the bacterial plasma membrane (Fernández et al., 2010; Lee et al., 2014), and there was a hope that the inhalation of colistin may be a reliable protective measure in adults (for children, in the light of its high toxicity, this is possible only after the child reaches 6 years age).

Recently, however, a new problem has arisen that may limit the use of colistin. A transmissible plasmid has been isolated encoding MCR-1, an enzyme that transforms the bacterial lipid A in the outer membrane to a colistin resistant state (Liu et al., 2015). This plasmid has been found in *Escherichia coli* strains, but given the relatively ease of interspecies migration for plasmids it is almost inevitable that a situation will arise when some strains of *P. aeruginosa* infecting patients with CF will acquire additional transmissible resistance to colistin. It is unclear yet corrected whether a strain with such a plasmid could be displaced by a more physiologically active but colistin-sensitive strain in the absence of the antibiotic selective pressure, as sometimes happens in the case of chromosomal resistance to colistin. Therefore, it is possible that the use of bacterial viruses – phage therapy – may not only be suitable, but also a valuable method of antibacterial therapy, especially in the treatment of children in specialized CF departments, under careful medical and microbiological control.

## Comparison of Bacteriophages and Antibiotics as Antimicrobial Agents: Evaluation of Results in the Application of Phage Therapy

Bacteriophages acquire the properties of living systems in the process of infecting sensitive bacteria. There are different viewpoints about the relations between phages and their hosts. They are often considered enemies of bacteria, but one can also look at them from the perspective of being a potential source of additional genetic material designed to maintain the evolution of the bacterial domain and their adaptive potential. In either instance, their natural purpose is not for use in the treatment of human and animal diseases Thus, it is evident the desire do not use as therapeutics the phages with substantial potential to transduce bacterial genes. It is necessary to bear this in mind in the application of phage therapy and this is one reason why the outcome of such treatment can be of an unpredictable nature. Additionally, we cannot preclude the possibility of events such as recombination with related phages, the appearance of different phage resistant bacterial mutants, or the establishment of pseudolysogeny (Hobbs and Abedon, 2016) in sensitive bacteria, and interactions with different plasmids, temperate, and virulent phages of unrelated species. Knowledge of possible modifications in relations of virulent phages and bacterial hosts is very important for success of phage therapy. That requires prior conduction of *in vitro* comparative studies of the growth of different phages infecting different clinical isolates in microbiotas.

So how do we evaluate the effectiveness of phage therapy? When treating open infected wounds using bacteriophages, even a single use of an appropriately selected phage mixture is sometimes sufficient (Voroshiliova et al., 2013) for successful cleansing of the wound. Objective assessment of antibacterial phage therapy in the treatment of pulmonary infection can be made by comparing the changes in bacterial compositions in the sputum of patients before and after phage application (cited in Abedon, 2015), with evaluation of the resistance of surviving bacteria to the therapeutic phages utilized (see later). This is important, as the emergence of resistant variants provides a level of confirmation as to the success of the previous phage inhalation treatment, but on the other hand, demonstrates the need to continue treatment with the selection and introduction of new active mixtures of phages. Sometimes the effect of phage therapy can only be observed after several days to allow the accumulation of an active phage concentration sufficient to infect most pathogenic cells. In addition, evaluation of the effectiveness of phage therapy may be valid only in conditions preventing cross-infection of the patient with other strains that are currently circulating in the hospital.

## Prerequisite for Safe Phage Therapy – Refusing to Expand the Lytic Spectrum of Phage Preparations With Random Bacteriophages

In Eastern European countries where phage therapy is officially recognized, the decision on its application is taken by medical practitioners. Factories located across Russia are producing phage preparations active against several regionally chosen species of pathogenic bacteria. With a certain periodicity, the phage preparations are going through a process of “adaptation” to novel circulating phage resistant strains derived from various specialized clinics and hospitals of the region by the introduction of new phages into them from external sources to overcome this resistance. This method of enhancing the lytic spectrum of phage commercial preparations is simple, fast, inexpensive, and yet very effective (Voroshiliova et al., 2013). Even though the newly introduced phages have not been identified and their properties remain unexplored, these preparations show good results in the short-term treatment of various open infected wounds, intestinal, and urinary tract infections. The composition of the phage preparations having a similar designation, but produced by different manufacturers at different times, may differ in their activities. This non-identical nature of phage preparations of varying origin can allow for performance comparisons to be made and subsequently allow one to choose the most appropriate mixture. Generally speaking, however, doctors only consider the possibility of utilizing phage therapy after repeated failures with different antibiotics (and even then not always!). Nevertheless, the use of such preparations of random enrichment, containing uncharacterized phages, including temperate, transposable and filamentous ones potentially involved in promoting the evolution of bacterial pathogens in CF can lead to undesirable consequences (McCallin et al., 2013).

In the course of treating chronic infections, the introduction of new active phages should take place in real time, in order to prevent the occurrence and accumulation of new phage-resistant variants. It is advisable to consider the possibility of modifying the composition of a medicinal mixture, in which the probability of occurrence of such undesirable effects could be eliminated or substantially reduced. This would help transform phage therapy from an almost forgotten in official medicine procedure with limited use into an established method for antibacterial therapy (beyond the realms of CF). First of all, it is necessary to prohibit the expansion of the lytic spectrum when introducing a mixture of new phages with unexplored properties. Then, it would also be desirable that the procedure for adaptation of activity of the phage mixture be applied to the treatment of an individual patient and that this is carried out within a clinical setting. Such modification does not require the introduction of significant organizational changes, and this work could be accomplished by existing professional staff of the microbiology laboratory at the clinic that possess some practical experience working with bacteriophages. Their task will be to monitor whether phage-resistant strains arise in patients and to then select new phages from a phage bank to restore the therapeutic activity of the phage mixture.

Since in the absence of active antibiotics, phage therapy could become a permanent clinical procedure, it is important to keep in mind that some unpredictability can arise that could affect the outcome of each phage application. Bacteriophage multiplication in lungs may depend on many uncontrollable conditions – interactions of different species of bacteria, the nature of the infected surface, lung fluid composition and viscosity, amongst other variables.

A critical factor in order to support uninterrupted treatment time is the permanent expansion of the lytic spectrum of phage preparations. Therefore, it is of the utmost importance that rigorous monitoring procedures are established so as to hastily detect potential phage resistant mutants, as their rapid eradication may be concentration dependent. Up until now, the presently adopted procedure for expanding the range of lytic activity is ineffective, as adaptation demands too much time. Optimization of lytic spectra for phage therapy, however, should be personalized in real time. Naturally, it not only increases the demands on the reliability of the active phage preparation, but also transforms clinical phage therapy in the ongoing research. As an example - in medical preparations temperate phage and their lytic variants, even those possessing unique lytic spectra, should be absent. Indeed, potential transitions to the prophage state (as a result of recombination with phages already present in the lungs) can result in the imparting of properties such as heightened pathogenicity, increased stability in the environment, and capacity for epidemic spread.

We believe that an absolute prerequisite for the implementation of phage therapy worldwide as a method of antibacterial therapy in CF is the investigation of genetic and other properties of phages administered into the preparations and potential interactions associated with these. One can expect that such work in prospect will provide valuable new data required not only for the characterization of phages as antibacterials but also, for example, as the carriers of genetic material capable of compensating the CFTR gene mutation responsible for CF.

Numerous studies have been carried out with the end goal of achieving the resurrection of phage therapy within the Western world, including the treatment of CF associated infections. Various models of acute and chronic infection in mice, rats, wax moths, and cell culture provide an excellent framework for testing phages under a variety of conditions. For example, it has previously been demonstrated that the use of wax moths (*Galleria mellonella*) as a model allows one to carry out rapid comparisons of phage activity, useful for making swift evaluations of effectiveness with respect to particular phage preparations *in vivo* during the treatment process (Beeton et al., 2015; Olszak et al., 2015). Using a mouse model, it is also possible to estimate bactericidal activity by lytic phages in lung infection. One study has shown a good correlation of activity *in vitro* and *in vivo* for all virulent phages utilized, however, with the exception of two species, shown as being insufficiently active *in vivo* despite good *in vitro* activity (Henry et al., 2013). This is a significant observation as it highlighted the need to increase the lytic activity in phages of several species through the selection of specific mutants displaying enhanced virulence. This also demonstrates the need to develop optimal configurations for phage mixtures, so as to achieve the best treatment outcomes. Indeed, with respect to the utilization of multi phage preparations, the therapeutic activity is a product of the combined effect of all phages present. In fact, Alemayehu et al. (2012) previously demonstrated the ability to stop a primary infection of mice with a multidrug-resistant *P. aeruginosa* strain taken from a CF patient with lung infection with such phage preparations. The bactericidal effect of these phages has been confirmed with an *in vitro* infection of this strain on a CF biofilm bronchial epithelial (CFBE41o) cell line. In addition to all of this, application of two strain specific virulent phages in a murine model of acute pulmonary *P. aeruginosa* infection was accompanied not only by rapid elimination of the pathogen, but by a concomitant decrease in the level of inflammation (Pabary et al., 2015). However, the requirement for continuous monitoring of lung composition in CF patients during the application of phage therapy is essential, as is the need to take additional measures in order to prevent the permanent adhesion of bacteria to the pulmonary epithelium. Friman et al. (2016) demonstrated that individual pre-adaptation of a phage for a different patient increases the efficiency of the phage killing effect. However, conducting this pre-adaptation process may take some time and delay the onset of the actual treatment, which is obviously not always possible. In light of all this research, it is clear that there is significant promise for the implementation of phage therapy in the treatment of CF associated infection and no doubt beyond this, however, rigorous protocols must be set in place if we are to ensure both the safety and maximum potential of treatment.

### Significance of Temperate Bacteriophages in CF Clinical Manifestations

Studies using metagenomic analysis have shown how temperate bacteriophages of *P. aeruginosa* in clinical conditions following induction become involved in the horizontal transfer of DNA, and acquire selective preference for the development and accumulation in the specific conditions of the lower lung (Tariq et al., 2015). Their ability for transfer of additional genes increases with time during the deterioration of patient lung function and disease prognosis. This represents the first, direct clinical confirmation for the proposed evolution of phages *in vivo* at mucous lung surfaces. The active role of phage gene expression in the course of the disease and its prognosis has been confirmed in a study involving the Liverpool epidemic strain. Other research (Lemieux et al., 2016) has shown that groups of strains possessing mutations in prophage regions and pathogenic islands displayed reduced pathogenicity in a rat model of chronic lung infection and was associated with disturbances in phage transcription.

### Critical Analysis of the Results of Phage Therapy in *Pseudomonas aeruginosa* Infections for a Group of Children With Cystic Fibrosis

The predominant goal of antibacterial therapy in CF is to cure chronic bacterial infections, especially those due to *P. aeruginosa*, with the aim of increasing patient life expectancy. This is of paramount importance in children up to 6 years of age, due to the inability to utilize colistin in extreme cases of multidrug resistant infection. Phage therapy in CF therefore should be implemented with this group in the forefront of our minds. The significance of the ongoing research will hopefully result in a resurgence of interest in phage therapy in the Western world, especially to support children suffering from CF.

**FIGURE 1:**
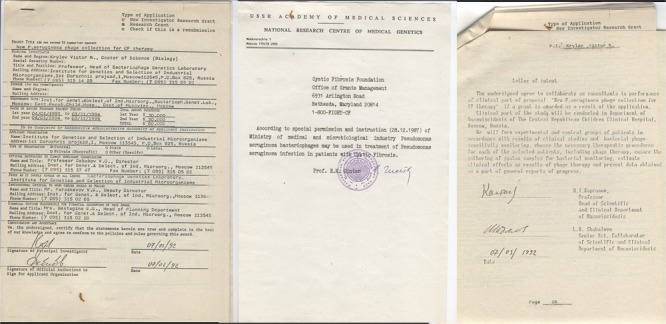
**Official permission for application of phage therapy in CF unit of The Central Republican Children Clinical Hospital, Moscow, Russia and agreement of physicians in CF unit to collaborate in the study (for request of grant support)**.

Of relevance is collaborative research that took place in 1991–1992 between the Laboratory for bacteriophage genetics (being at that time in the Institute for Genetics and Selection of Industrial Microorganisms, Moscow, Russia), and the Department of Mucoviscidosis (CF) in the Children’s Republican Clinical Hospital in Moscow. Following the acquisition of parental consent and permission from the Academy of Medical Sciences (**Figure 1**), a study was conducted into the use of *P. aeruginosa* phages in the treatment of five children with CF. Whilst there were no specific restrictions for the number of children to be involved in this research, the need to limit the number of participants was felt as being necessary due to the fact that this was the first trial involving the inhalation of phages for the treatment of CF associated infection and prudence is always wise in such uncharted territory. In addition, the phage preparations used were tailored and prepared in highly concentrated forms with purification on cesium chloride density gradients for participants to use over 7–10 days. Due to these rigorous protocols, laboratory resources were limited to a small number of patients.

The five children were aged from 7 to 12 years old. The compositions of the *Pseudomonas* strains in their lungs were studied. The differentiation of strains was performed by taking into account features such as resistance to clinically used antibiotics, mucus (alginate) production, colony pigmentation, temperate phage production, and sensitivity to various bacteriophages (including temperate) available at that time in the laboratory collection. Usually at least two different *P. aeruginosa* strains were found in the sputum of each child. The different mixtures of virulent bacteriophages were prepared for each child based on testing bacterial strain sensitivity to phages. The phages utilized were chosen among several species classified at the time, including phiKZ-, Lin68-, and PB1-like phages, and those of a group referred to later as the phiKMV-like phages. The phages were selected for each child based on the greatest lytic effect on strains isolated from sputum (those which resulted in transparent lysis in the spot assay). Selected phages were grown overnight in petri dishes with *P. aeruginosa* PAO1 by confluent lysis, followed by resuspension into saline and bacterial debris removed by centrifugation. The supernatant was treated with chloroform to eliminate residual bacterial cells and phages were further concentrated by polyethylene glycol precipitation. The resulting phage were then subjected to centrifugation in a cesium chloride gradient. Following dialysis, individual preparations were mixed with titers of at least 10^11^–10^12^ particles/ml. Such high titres were used based on the assumption that a higher concentration of phages in inhaled mixtures would allow the determination of an effect (or lack of) in a short period of time. However, during the actual clinical application of the phage mixtures the preparations were diluted several times as it was found that concentrated mixtures were unable to pass through the inhalation device. The inhalations were carried out over the course of a week along with the standard monitoring protocols of the children’s status.

It was found that one child had gone through a short period involving a transient increase in body temperature. The others had no obvious changes in their general condition. In two cases, comparison of bacteria in sputum samples after the course of phage therapy revealed evident changes attributable to the use of phages. In one of the sputum samples, were prevailing phage-resistant variants displaying the appearance exhibited by the original pigmented bacteria and the typical mucoid properties observed in *P. aeruginosa*. In the sputum of the second child were phage-resistant variants of *P. aeruginosa* exhibiting novel properties. It was proposed that in this second case the original phage sensitive bacterial cells were substituted for another phage-resistant strain, with the new properties, possibly due to cross-infection from other patients.

The emergence of phage resistant variants of the initial infecting bacterial strain in one child and the substitution of sensitive bacteria for a new phage resistant strain in the lungs of the second child is clear evidence as to an effect from phage therapy *in vivo*. The absence of detectable changes in three other children could be attributed to various reasons such as too low a concentration of phage mixture following dilution of the original formulation, the high viscosity of mucus hindering the spread of the phage, and insufficient duration of phage application. These results were presented in several CF meetings and were considered as initial evidence for the safe use of phage therapy in CF (Shabalova et al., 1993, 1995). Unfortunately derivative studies were unable to be carried out at the time due to insufficient funding opportunities. However, giving the growing antibiotic resistance, this is starting to change. The use of commercial preparations of phages in the treatment of *P. aeruginosa* infection in children with CF in Georgia also has shown some promise (Kutateladze and Adamia, 2010).

## The Modular Composition Principle for the Preparation of Therapeutic Phage Cocktails

The pediatric study described above showed that personalized phage preparations can have a noticeable effect on a brief timescale. The previously active *P. aeruginosa* strains in lungs of two children were eliminated and were substituted with other strains. Independent of their origin (resistant mutant or cross infection), such situations require immediate re-evaluation of the therapeutic mixture for each of the two children showing positive reactions. The use of commercial preparations containing uncharacterized isolates is not an appropriate course of action. Therefore, a series of rapid protocols needs to be set in place to permit the rapid isolation, characterization and choice of phages and to allow the appropriate modification of preparations within a therapeutic timeframe. All of this must to be carried out in a safe and pragmatic manner.

We propose a method to enhance the rate at which phage mixtures are prepared for personalized therapy in CF through an alternative approach that will also permit the enhancement of the lytic spectra of the resulting preparation. The fundamental idea is to utilize what we refer to here as a modular principle. These modules may be monospecies phage mixtures or a mixture of phages of unrelated species taken from a collection of pre-selected phages. The advantage of this approach is that the expansion of phage activity of such mixtures has not been achieved through a one-time introduction of unknown phages in the final product, but introduction into each such modular product, a new phage of the same species but with enlarged activity for monospecies mixtures or some another new phage into a heterospecies mixture. In a joint multiplication of such mixtures, closely related phages may recombine, potentially resulting in progeny exhibiting novel spectra of lytic activity.

Thus, to transition to this novel approach we propose (1) to select a limited number of well-studied phage groups, containing a great deal of described non-identical phages and compose from these phages monospecies mixtures displaying unique lytic spectra; (2) merge, into a common single multispecies blend, well studied bacteriophages of other known species, with unique spectra of lytic activity; (3) Each of the preparations are propagated through infection of *P. aeruginosa* PAO1 or other acceptable strain; (4) The final therapeutic preparation for personalized therapy is composed of individual mixtures (modules) to allow for maximal lytic activity.

Obviously, the extensive accumulation and study of phages for each of the chosen species exhibiting a wide spectrum of lytic activity is an absolute prerequisite for the operation of this protocol. As described above, even short-term use of mixtures of constant composition from a small number of phages leads to the selection of resistant bacteria. Up until now there are only individual studies on the frequency of phages capable of infecting different bacterial species. For example, there is a report describing phages capable of infecting the bacterial species *P. aeruginosa* and *Burkholderia cepacia* (Nzula et al., 2000). But it is important to consider and evaluate potential interspecies transduction in such instances. In another study (Malki et al., 2015), the authors demonstrate that PB1-like phages of *P. aeruginosa* isolated from a natural habitat can infect unrelated species of the *Arthrobacter, Chryseobacterium*, and *Microbacterium* genera. It also suggests the possibility of interspecies transduction with potentially unpredictable results. Of course, despite this possibility, this is a natural inevitability associated with bacteriophages, and is independent of the phage therapy process (Penadés et al., 2015). Complete prevention is therefore not a realistic goal. However, we believe that additional studies are necessary to gain an insight into the effect of horizontal gene transfer with *in vivo* applications of phages.

**FIGURE 2:**
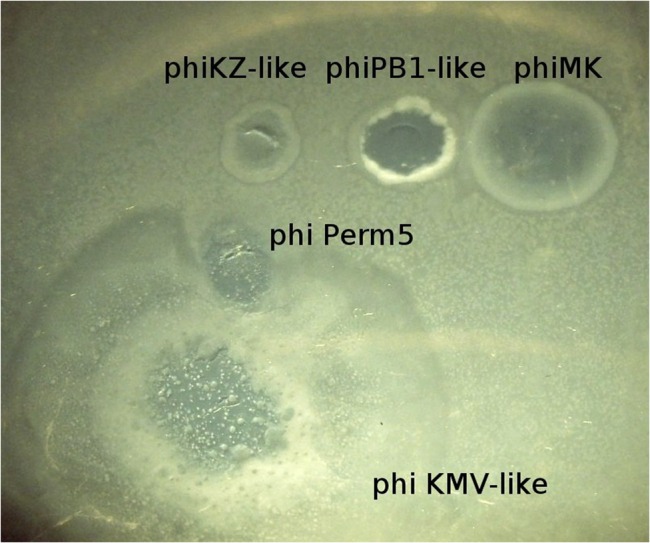
**Different growth appearance on *P. aeruginosa* PAO1 lawn permits preliminary identification of five bacteriophage species**.

Of critical significance in the selection of phage species for treatment of pseudomonade infection in CF is the ability to infect different natural *P. aeruginosa* strains. From a practical standpoint this is achieved through the recognition of such susceptible strains through the appearance of plaques. Moreover, in primary pulmonary infections of patients with CF, any strain of *P. aeruginosa* is capable of participation. As a general dogma, in the process of producing future proof therapeutic mixtures, the best species of phages form the foundation upon which refresh cycles take place using new modules to expand the overall lytic activity as necessary. Based on these general considerations and prior experience, we have chosen three phage groups suitable for composing integral monospecies phage mixtures as a foundation: phiKZ-like, phiKMV-like, and PB1-like phages. Their frequent occurrence in current commercial mixtures may reflect the broad spectrum of their lytic activity. In addition, they are well characterized – many genomes of phages in these groups have been sequenced and annotated. Finally, phages of these species produce recognizable plaques (**Figure 2**) making it easy to identify them amongst mixed populations in natural samples. There are, however, other phage species which seem promising for additional monospecies phage mixtures and which form the basis for subsequent research.

## Features of Species Selected for Monospecies Phage Mixtures and Choosing the Best Sources of These Phages

**Table 1** lists the general characteristics of a group of selected virulent phage species active on *P. aeruginosa* (Adams et al., 2015). The function of most gene products of these phages remains unknown. Therefore, the choice of these phage species for therapy is based mainly upon the results of long-term work in several laboratories and, in addition, on the frequent presence of these phages in commercial therapeutic mixtures, from the days of D’Herelle. It is possible to assess the efficiency of different phages by determining their proportionality following a therapeutic application using plaque phenotype and PCR and then appropriately adapt the mixture for the next round of phage therapy. Below is an overview of the major groups of phages proposed to be utilized within monospecies and heterospecies mixtures.

**Table 1:** *Pseudomonas aeruginosa* phage species, proposed for use in phage compositions as mono- or heterospecies mixes.

No	Subfamily and/or genera	Phage family	Representatives of phage species in our collection (N_o in NCBI)	The number of phages in the database NCBI on April 2016	Genome size Min – Max bp	The number of ORFs	The number of ORFs with known function	Our collection
1	Phikmvlike viruses	Podoviridae	phiKMV (NC_005045)	14	42351 – 43639	47–56	Max 28	phiKMV, phiNFS (+8 phages)
2	Pbunalike virus	Myoviridae	phiPB1 (NC_011810)	29	64144 – 68871	85–97	Max 33	phiPB1, F8, SN, 14/1 (+9 phages)
3	Phikzlike viruses	Myoviridae	phiKZ (NC_004629)	2	266743-280334	306–333	Max 144	PhiKZ (+17 phages)
4	Luz24like viruses	Podoviridae	phiTL (NC_023583)	10	44030 – 45808	58–73	Max 28	phiTL, phiCHU
5	Phikzlike viruses	Myoviridae	phiEL (NC_007623)	1	211215	201	21	phiEL, phiCHE, phiRU
6	Felixounavirinae PAKP1like virus	Myoviridae	phi MK (KU761955.1)	25	83598 –94555	149–188	Max: 28	phiMK
7	N4like virus	Podoviridae	PhiPerm5^∗^	13	72028 –74901	83–115	Max: 26	phiPerm5
8	Phikzlike viruses	Myoviridae	phiLin68^∗^	N/A	N/A	N/A	N/A	phiLin68, phiLBG22

*^∗^Genome is not sequenced.*

### The phiKZ-Like Viruses

The phiKZ-like group of giant phages infecting *P. aeruginosa* includes several species and are of interest not only because of their use in a therapeutic setting, but also as a unique model for the study of phage evolution and specific packaging of the genomic DNA (Burkal’tseva et al., 2002; Mesyanzhinov et al., 2002; Krylov et al., 2003, 2004, 2005, 2011; Hertveldt et al., 2005; Shaburova et al., 2008; Pleteneva et al., 2010; Sokolova et al., 2014). This group of *Myoviridae* phages includes several phage species that exhibit common properties of the type member phiKZ (particle size and morphology, the presence of an inner body in the capsid, and a specific packaging mechanism of phage genomic DNA). In sequenced and annotated genomes of two different species of this group – phiKZ and phiEL, genes encoding a DNA polymerase have not been found. This may prove to be a common feature for this group. The genomes of PhiEL and phiKZ differ in their sizes and lack any tangible homology at the nucleotide level. Two other phages closely related with phiEL are phiRU (isolated from soil) and phiCHE (from a clinical strain of *P. aeruginosa* isolated from an infected burn wound). phiEL-like phages may be crossed in different combinations, giving rise to viable progeny. This is potentially useful for generating recombinants displaying variable host specificities both *in vitro* and *in vivo*.

**FIGURE 3:**
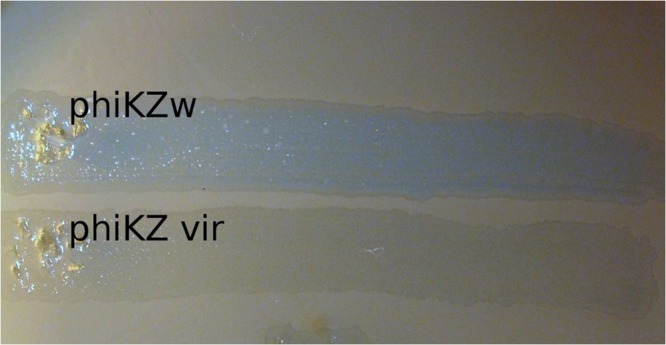
**The vir-mutant of bacteriophage phiKZ is unable to enter the pseudolysogenic state**.

**FIGURE 4:**
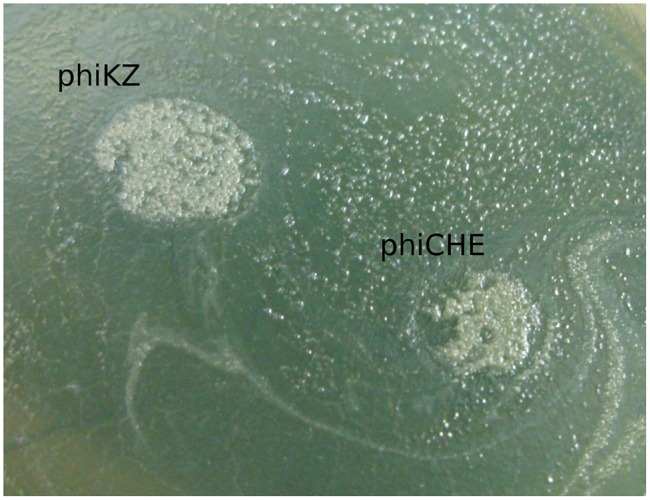
**The growth of phiKZ and phiCHE pseudolysogens in places of phages application on the background of auto-lysed lawn of plaque forming clinical isolate *P. aeruginosa* CF017 after prolonged incubation**.

Phages phiLin68 (of Lindberg phage collection) and phiLBG22 (our lab collection) are representatives of the third group in the phiKZ-like viruses. These phages were included due to the fact that they can lyse some strains resistant to phages from the phiKZ and phiEL groups. We have found non-identical phiKZ-like bacteriophages in all therapeutic commercial mixtures from different manufacturers. Interestingly, phiEL- and phiLin68-like phages have not yet been found within therapeutic mixtures. In creating therapeutic preparations containing phiKZ-like phages, one should bear in mind that all species in this group have the ability to transfer bacterial cells into persisting (“pseudolysogenic”) state (Pleteneva et al., 2010). It has similarity with effect of lysis inhibition after infection of *E. coli* bacterial cells with wild type phage T4 but not with r-type mutants (rapid lysis) (Doermann and Hill, 1953). A possible reason may be the maintenance of certain structures in cell envelope after the re-infection of bacteria with wild-type phage (Krylov, 1970). Meanwhile phiCHE bacteriophage, closely related with phiEL, has been isolated from a bacterial strain in burn wound. Different mutations in genomes of phiEL-like phages influence the effect (Krylov et al., 2011). This is manifested through the appearance of opalescence of bacterial growth (**Figure 3**) and a significant increase in the final yield of phage. In this state, however, the lysis of infected bacteria is delayed. For this reason, it is not desirable to use wild type phiKZ-like phages as components in therapeutic preparations for the treatment of *P. aeruginosa* pneumonia. In addition, amongst *P. aeruginosa* strains isolated from the lungs of CF patients are often those strains capable of producing autoplaques (Pillich et al., 1985). We have found that *in vitro* infection of clinical isolates in CF with different wild type phiKZ-like phages leads to a decrease in the level of autoplaquing of these bacteria. As a result, the growth of phiKZ-like pseudolysogenic bacteria occurs more rapidly than the growth of the original uninfected bacteria (**Figure 4**). Therefore, to maintain the possibility of using the wide lytic potential of phiKZ-like phages in CF in a safe manner, we have isolated a group of mutants in phages phiKZ and phiEL which have lost the ability to transfer bacteria into the pseudolysogenic state (Krylov et al., 2011). Some of these mutants are also showing signs of hypervirulence, capable of lysing pseudolysogenic bacteria. We believe that mutant phages such as these, belonging to the phiKZ- like group can be used safely as part of the therapeutic mixtures, complementing each other’s activity and thus providing us with an additional weapon in our arsenal.

The decision to include phages of Lin68 species into a therapeutic mixture could be made after the isolation of similar mutants and (preferably) after the sequencing and annotation of their genomes, as done for phiKZ and phiEL.

### PhiKMV-Like Bacteriophages

**FIGURE 5:**
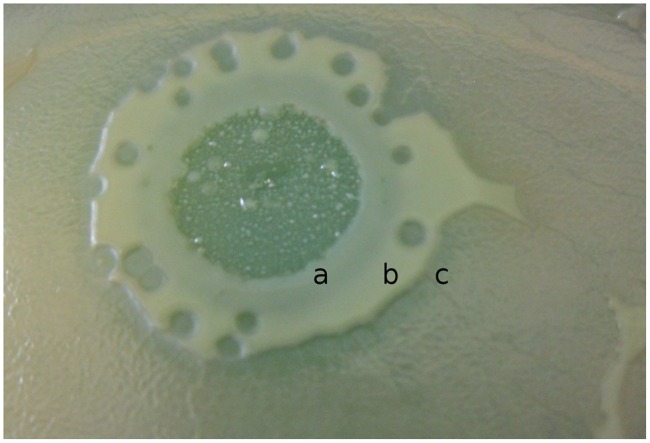
**The growth of KMV-like phage phiNFS on lawn of plaquing *P. aeruginosa* clinical isolate inhibits plaque formation**. The different activities of diffusible products can be observed: **(a)** complete inhibition of plaquing; **(b)** a significant reduction in the size of the plaques to a certain size; **(c)** an abrupt discontinuation of interaction. This is an example of a phage–host interaction which can seriously influence the results of phage therapy by enhancing bacterial growth in lung biofilm. Only specially prepared phage mixture can prevent undesirable effects of mono phage therapy.

**FIGURE 6:**
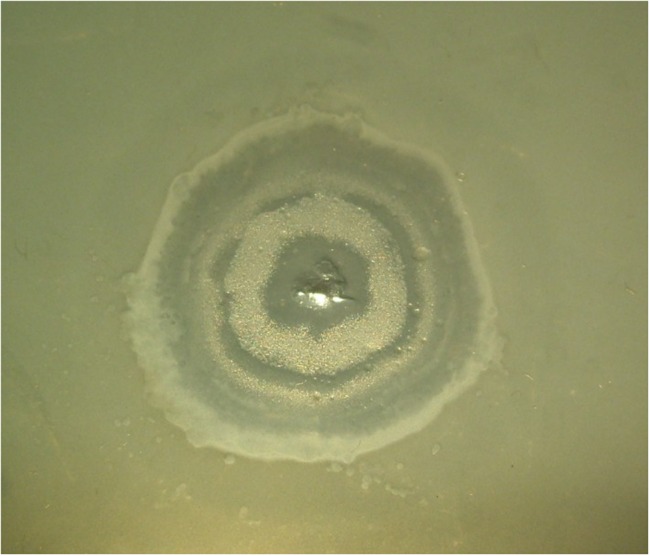
**The continuing phiKMV-like phage growth going through cyclic periods of lytic growth and pseudolysogeny**.

phiKMV – related bacteriophages (Lavigne et al., 2003, 2006; Burkal’tseva et al., 2006) are also frequent components of the various commercial mixtures and display different host ranges and plaque sizes to that of other groups. For example, a new phiKMV-like phage, phiNFS, has been isolated as a mutant from a commercial mixture by our group. The parental phage forms small transparent plaques on the lawn of strain PAO1 and in each such plaque mutants with increased growth rate have arose at high frequency (Krylov et al., 2015). The possible reason for this effect may be related with the use of a bacterial host exhibiting properties that differ from those in *P. aeruginosa* PAO1 in the production of the commercial mixture. Mutants isolated from various plaques may vary in growth rate on different clinical isolates. The ability for permanent modifications of different growth characteristics is an inherent feature of the phage phiNFS. The mechanisms underpinning this are under investigation. Preliminary data suggest that it may be related to processes involved in quorum sensing. For example, in some clinical isolates phage phiNFS specifically inhibits the formation of bacterial plaques with a clear border of inhibition observed (**Figure 5**), which can be explained with the production in plaque growth of an unknown product which interacts specifically with cells of the plaque producing bacterial strain (“all or nothing”). This phage displays the greatest expression of a peculiar feature of the phiKMV-like phages: continuous (5–7 days) growth on aged bacterial lawns, something uncharacteristic of other species of *P. aeruginosa* phages. The cyclic nature of this growth may be due to the ability of the phage to overcome the physiological changes in bacteria arising as result of the aging process (**Figure 6**).

### PB1-Like Bacteriophages

**FIGURE 7:**
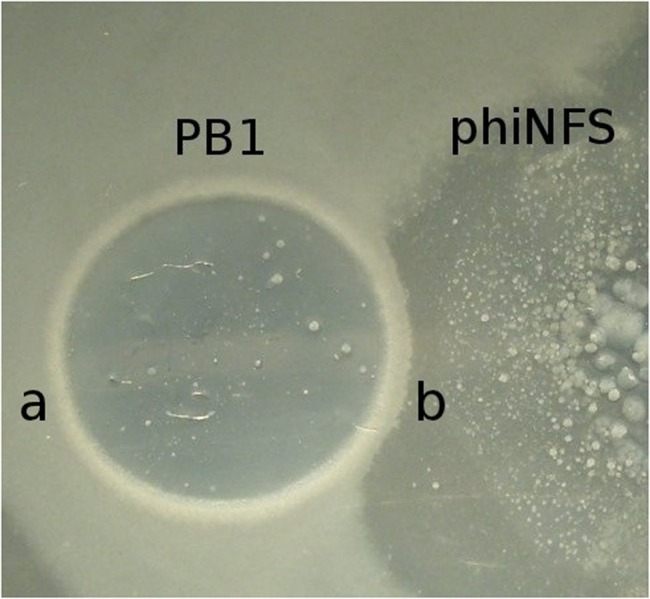
**The common feature of all PB1-like phages are specific bacterial bands bordering their plaques**. Such bands contain special bacterial cells in the pseudolysogenic state and which limit the growth of phages of other species. In some cases this property can influence the result of phage therapy **(a)** The growth of cells in pseudolysogenic state around phiPB1 phage plaque. **(b)** Interaction of lytic areas of bacteriophages phiPB1 and phiNFS.

PB1-like bacteriophages are another frequent representative of phage cocktails. A clear plaque with a characteristic feature - a dark stripe along the edge of the growth (**Figure 7a**) is a typical feature of the group and may be used as a species characteristic in looking for new phages of the group. This stripe appears at the end of plaque growth and it consists of infected cells in a specific state (Krylov et al., 2015). It limits the spread of the plaques of other phage species (**Figure 7b**) but is unable to prevent penetration of phage particles into PB1 plaques. Advantages of the PB1-like phages are in the simplicity of their selection from enrichment of therapeutic mixtures (non-identical phages can be often be found in samples taken from the same natural water sources), a wide range of lytic activity, and a relatively low frequency of phage resistant mutant generation (Garbe et al., 2010).

### Heterospecies Phage Cocktail

**FIGURE 8:**
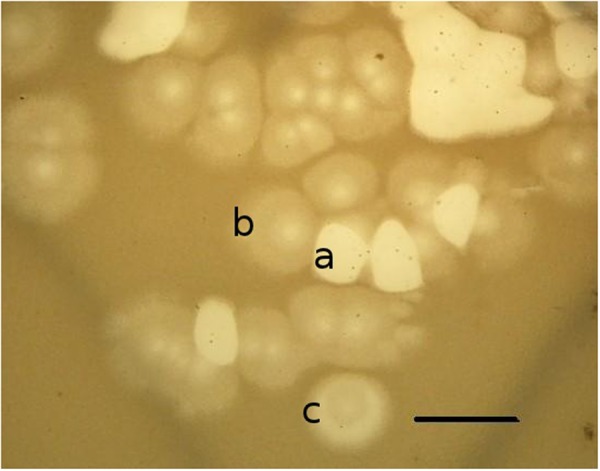
**Diversity of bacteriophage phiCHU plaques on lawn *P. aeruginosa* PAO1 (pMG53).** The replanting of phages from clear **(a)**, turbid **(b)** or semiclear **(c)** plaques again leads to production of all types of plaques. Bar is 1 cm.

Besides the three monospecies mixtures, we suggest the use of an additional mixture comprising phages of several unrelated species. Each of these phages display certain differences in their spectrum of lytic activity, for example, the ability to lyse bacteria exhibiting resistance to phage mixtures of the three previously mentioned major species, as well as strains carrying a plasmids conferring phage-resistance. This group includes phages phiEL (NC_007623), phiLin68, phiPerm5, phiMK (KU61955), and phiCHU (NC_028933), representing several different groups. As all the phages of those selected for this mixture are relatively easy to recognize (Krylov et al., 2015) the choice of new phages for the purpose of expanding the lytic spectrum will face little in the way of problems. Nevertheless, it is of critical importance to pay special attention to the possibility of significant differences existing between closely related phages. Sometimes their genomes can contain small variations which result in them being inappropriate for therapeutic use. For example, phages phiTL and phiCHU showed good growth on PAO1 in the presence of IncP2 group plasmids (for example, pMG53 plasmid) and on some clinical isolates resistant to other phages. The improved growth of phages is accompanied with occurrence of unstable plaque morphology (**Figure 8**). However, only phiCHU can be utilized in therapeutic mixtures as the phiTL genome was found to encode a putative transposase fusion gene (NC_023583, Magill et al., 2015). These phages are closely related to LUZ24 (Ceyssens et al., 2008; Wagemans et al., 2015) and phiPaP3 (Tan et al., 2007), together belonging to the LUZ24likevirus genus.

## *P. aeruginosa* Pao1: The Best Host for the Study of New Phages, But Not for the Multiplication of Therapeutic Mixtures?

In some cases, problems may arise when trying to find a suitable strain for the amplification of finalized therapeutic mixtures. One such problem is that *P. aeruginosa* strains often contain the prophages of filamentous phages. The reason for the frequent occurrence of filamentous prophages is believed to be that they provide adaptive capabilities to *P. aeruginosa* under unfavorable conditions (Rice et al., 2009). The accumulation of filamentous phages within the mucus of the lungs promotes crystallization and establishment of solid biofilms (Secor et al., 2015). Therefore, choice of hosts containing these prophages (such as *P. aeruginosa* PAO1) can result in the unintentional inclusion of filamentous phages into the final preparation. The therapeutic use of a phage mixture obtained as a result of random enrichment and amplification on an unstudied host strain is therefore undesirable. In addition, one should note that despite the fact that the PAO1 strain of *P. aeruginosa* is a well studied and long standing work horse of many microbiology laboratories, one should be careful with respect to its potential use in the final amplification of phage preparations. This is due to the fact that it possesses a fairly high pathogenic potential, as seen by its long term presence within the lungs of mice, though it is advantageous for the prevention of introducing temperate phages with undesirable features (Tayabali et al., 2015). It does, however; remain as the accepted strain for the study of the phages themselves.

The work on the creation of a reserve collection of new phages for inclusion into monospecies phage mixtures should be carried out on an ongoing basis. In accordance with our experience, the selection of new phages and reproduction of monospecies mixtures would be better accomplished with the strict use of the standard strain of *P. aeruginosa* PAO1 and its minor variants (e.g., those carrying plasmids for propagation of plasmid dependent phages such as phiCHU). The use of the standard strain PAO1 is necessary for preventing the introduction of potential temperate phages with undesirable features (for example, transposable capabilities) into therapeutic phage mixtures. It is evident, joint multiplication of non-identical but closely related phages can and will produce valuable recombinants, including ones with an increased spectrum of lytic activity. Thus the simple act of reproduction of a monospecies mixture will create an additional opportunity to expand the lytic spectrum.

## “Non-Essential” Genes and the Prospect of “Correcting” the Genomes of Bacteriophages

We are confident that phage therapy of *P. aeruginosa* infection in children with CF is a very promising approach and is by no means limited to the use of natural phages from well-studied groups. It is likely that novel methods for genome editing will be utilized in the enhancement of therapeutic phages. This will permit less time consuming selection procedures for phages exhibiting novel lytic spectra. It may therefore be of some interest to investigate the reasons behind significant differences in genome sizes of phages exhibiting identical morphologies, due to the presence in the genomes of some phage species, regions which contain potentially non-essential genes with accessory functions. Such phages could be used as convenient vectors. For example, in phages phiKZ and phiEL which belong to different but phylogenetically related groups of giant phiKZ-like phages, we observe that phage particle sizes and the internal volume of the capsid are similar. The genome of phiEL, however, is ∼80 kb less than that of phiKZ. The cause of this difference may be, for example, a discrepancy in evolutionary rates of the morphological structure of the phage particle and its genome size. One must also consider that increases in genome size are limited by the capacity of the phage particle, so it could be reasoned that evolution would favor smaller genome sizes due to physical constraints. The viability of phage particles (in particular, the ability of DNA be injected into cell) though, may depend on the density of DNA packaging, and the loss of a significant number of genes, even those whose functions have ceased to be significant in the development of the phage, could prove lethal. In such cases there are two different strategies that may permit phage survival. One such strategy would be the evolution of a novel DNA packaging mechanism. Another possibility is via the conservation of genes whose functions become unnecessary or to replace them by similarly sized DNA sequences of temporal origin. As it happened, both of these strategies have been implemented. In the case of phage phiEL a variable packaging mechanism has arisen, which provides the necessary density for DNA packing and thus injection (Sokolova et al., 2014). Another postulated mechanism can be illustrated by comparing the genomes of two closely related phages of *P. aeruginosa* PAO1 – temperate phage phiD3 and its naturally lytic variant phiPMG1 (Krylov et al., 2012). In the central part of the genome of these phages there exists a large region containing gene fragments of variable origin. The presence of extensive areas of insignificant DNA (“genome gaps”), is an apparently common phenomenon. They were also found when comparing of the genomes of two transposable phages, PM105 and B3 (Pourcel et al., 2016).

Phages which possess these genome gaps could form a solid basis for the directed design of therapeutic phage genomes, for instance to insert genes that increase the efficiency of phage lysis. For example, the insertion of genes encoding bacteriocins, capable of killing bacteria of other pathogenic species, usually associated with CF. This idea is extremely promising but will require not only knowledge of the detailed functions of all genes and their interactions, but also evidence that the introduction of new genes will not result in adverse unwanted effects. Also, development and use of such future artificial phages will require thorough study of their potential interactions with the natural microbiota in humans and their potential environmental impacts.

### Expanding the Collection of Therapeutic Phages: National and International Phage Bank Cooperation

The implementation of continuous personalized phage therapy will require a cooperative and efficient system to allow the exchange of specific bacteriophages. The existence of a central phage bank keeping most of the phages active on *P. aeruginosa* and other pathogens active in CF and ensuring their availability for clinical laboratories in different countries would become an extremely valuable resource in addition to national collections. The basic purposes of this central phage bank would be:

(1) The accumulation of *P. aeruginosa* phages, their classification and comparison of their specific activities;
(2) Storage of phages which have been certified as safe;
(3) Composing initial monospecies phage mixtures with wide host range to adapt mixtures for real time requirements in hospitals;
(4) The delivery to hospitals of small samples of different phages to help clinicians and laboratory staff carry out the rapid selection of appropriate phages.

The understanding for the necessity of forming such phage banks is now generally accepted. Such institutions will form the central hub from which phage therapy can be developed.

## Conclusion

One of the purposes of this review was to show that choosing phages suitable for therapeutic use requires not only genomics and bioinformatics approaches, but long-term preliminary studies of their properties and their manifestations under different conditions in order to optimize their activity. The other purpose was to introduce a novel approach for the composition of therapeutic mixtures based upon a modular assembly of personalized preparations utilizing combinations of mono- and hetero- species mixtures to use in the treatment of *P. aeruginosa* infections in CF patients.

There is no doubt as to the efficiency of phage therapy when the correct selection of phage has been achieved. As discussed, this has been confirmed in experiments involving the eradication of *P. aeruginosa* lung infection in mice following intranasal administration of virulent phages (Alemayehu et al., 2012; Henry et al., 2013). There is also indirect confirmation of a positive response to phage activity under clinical conditions following from the observation (James et al., 2015) whereby authors found a correlation between increases in the number of free temperate phage particles in the sputum and improvements in the patient’s condition. This is understandable, because infection of bacterial cells with temperate phages usually results in the lytic cycle being the predominant mode of phage development. However, this study does not support the deliberate use of temperate phages. Finally, an example of the direct use of personalized phage mixtures in clinical practice described here reinforces the overall safety of the procedure itself.

Thus, we believe that phage therapy in CF is promising, but its use should be limited to those special cases whereby all available antibiotics have been proven to be ineffective. The group of potential patients includes children up to 6 years of age infected with multi-resistant strains of *P. aeruginosa* (as the use of colistin is not yet permitted) or adult patients infected with *P. aeruginosa* resistant to all available antibiotics, including colistin. As a matter of fact the transition to the permanent use of phage therapy, even in special cases, will require a significant change in the workings of specialized clinics. There must be special measures in place to separate the patients under phage therapy from others so as to prevent cross infection with other *P. aeruginosa* strains. The best solution would be the organization of a special center of phage therapy for CF patients, providing necessary resources and the capability to support and enlarge phage collections, etc. The work of personnel in such centers will include procedures utilized on a daily basis in academic laboratories. The aim of specialized teams of microbiologists in hospitals working with phages will be to monitor the changes in the proportion of bacteria resistant to used phages, developing and scheduling the introduction of new phage preparations, creating new combinations of compounds that extend the lytic spectrum, all procedures that should ensure the continuity of phage therapy. We believe that if certified manufacturers of commercial phage mixtures were to participate in the preparation of proposed mono- and heterospecies phage mixtures, it would both simplify and greatly in diminish the response time in the preparation of personalized cocktails and thereby help phage therapy become established as a major weapon in our arsenal against bacterial infection.

## Author Contributions

VK: corresponding author, organization of the work, writing of the article. OS: preparation of pictures, editing of text. EP, OP, and EC: selection and testing of phages, composition of phage mixtures, data analyze. MB, SK, and AK: DNA analysis of phages different species. LK and DM: DNA sequence of new phages, analysis of data, editing and correction of text.

## Conflict of Interest Statement

The authors declare that the research was conducted in the absence of any commercial or financial relationships that could be construed as a potential conflict of interest.
